# Association rare d’un hématome sous-périosté de l’orbite et d’un hématome extradural frontal: à propos d’un cas

**DOI:** 10.11604/pamj.2022.41.219.26914

**Published:** 2022-03-16

**Authors:** Mahamadou Aminou Sanda, Aminath Kelani, Addo Guemou, Samuila Sanoussi

**Affiliations:** 1 Service de Neurochirurgie, Hôpital Général de Référence, Niamey, Niger,; 2 Service de Neurochirurgie, Hôpital National de Niamey, Niger,; 3 Faculté des Sciences de la Santé, Université Abdou Moumouni, Niamey, Niger

**Keywords:** Traumatisme crânien, exophtalmie, hématome extradural, hématome sous-périosté de l’orbite, cas clinique, Head injury, exophthalmos, extradural hematoma, subperiosteal haematoma of the orbit, case report

## Abstract

L´hématome sous-périosté de l´orbite associé à un hématome extradural frontal est très rare. Nous rapportons un cas pris en charge dans notre service et faisons une revue de la littérature. Il s´agissait d´un garçon de 15 ans, victime d´un traumatisme crânien par arme blanche, une semaine avant son admission aux urgences. Il présentait une exophtalmie gauche inflammatoire et douloureuse, associée à une ophtalmoplégie et une cécité gauches. Le scanner cérébral montrait un hématome extradural frontal gauche associé à un hématome sous-périosté de l´orbite ipsilatéral. Une craniotomie frontale associée à une orbitotomie fracturaire a permis l´évacuation de l´hématome extradural puis de l´hématome sous-périosté. L´évolution clinique était favorable. La survenue simultanée d´un hématome extradural frontal et d´un hématome sous-périosté de l´orbite est extrêmement rare. Généralement ce sont l´exophtalmie et les troubles visuels qui attirent l´attention. Le scanner cérébral sans injection de produit de contraste réalisé en urgence permet de poser le diagnostic. Le pronostic étant essentiellement visuel, une prise en charge adéquate permet de sauvegarder l´œil et la vision.

## Abstract (en)

Subperiosteal haematoma of the orbit associated with frontal extradural hematoma is very rare. We here report a case treated in our Department with a literature review. The study involved a 15-year-old boy, victim of head injury from white weapon, a week before his admission to the emergency room. He had inflammatory and painful exophthalmos on the left side, associated with left ophthalmoplegia and blindness. Brain scanner showed left frontal extradural hematoma associated with subperiosteal hematoma of the ipsilateral orbit. Frontal craniotomy associated with fracture orbitotomy allowed evacuation of the extradural hematoma, and then, of the subperiosteal hematoma. Patient´s outcome was favorable. Simultaneous occurrence of frontal extradural hematoma and subperiosteal hematoma of the orbit is extremely rare. Generally, attention is drawn by exophthalmos and visual disturbances. Emergency brain scan without contrast agent injection can be used to make a diagnosis. Prognosis depends on visual function, then adequate management helps to safeguard the eye and vision.

## Introduction

L´incidence de l´hématome extradural représente environ 0,2-6% des complications du traumatisme cranio-encéphalique. Il est rare dans sa localisation frontale et sous frontale et les manifestations cliniques sont souvent pauci-symptomatiques [1,2]. L´hématome sous-périosté de l´orbite, également rare, constitue une entité clinique bien définie qui se manifeste par des symptômes ophtalmologiques: l´exophtalmie et les troubles visuels [1,2]. Leur association est exceptionnelle, car très peu de cas ont été rapportés dans la littérature [2,3]. La prise en charge doit être adaptée pour éviter l´évolution vers des séquelles gaves. Nous rapportons une nouvelle observation avec une revue de la littérature.

## Patient et observation

**Information sur le patient**: il s´agissait d´un garçon de 15 ans, vivant en milieu rural, sans aucun antécédent notable, victime d´un traumatisme cranio-facial après une agression par arme blanche, admis, 6 jours après, aux Urgences Chirurgicales de l´Hôpital National de Niamey pour une exophtalmie avec baisse de l´acuité visuelle du côté gauche et une désorientation temporo-spatiale.

**Résultats cliniques**: on trouvait un patient conscient, mais désorienté et agité, présentant une exophtalmie gauche douloureuse non pulsatile. Il existait un déplacement du globe oculaire gauche vers le bas, une ophtalmoplégie complète, un chemosis avec inflammation de la conjonctive. On notait également une ecchymose palpébrale et des excoriations frontales du même côté. Pour l´acuité visuelle le patient affirmait qu´il ne voyait rien à gauche mais qu´il pouvait percevoir la lumière. Les réflexes photomoteurs direct et consensuel étaient présents. L´examen neurologique était strictement normal.

### Evaluation diagnostique

**Figure 1:**
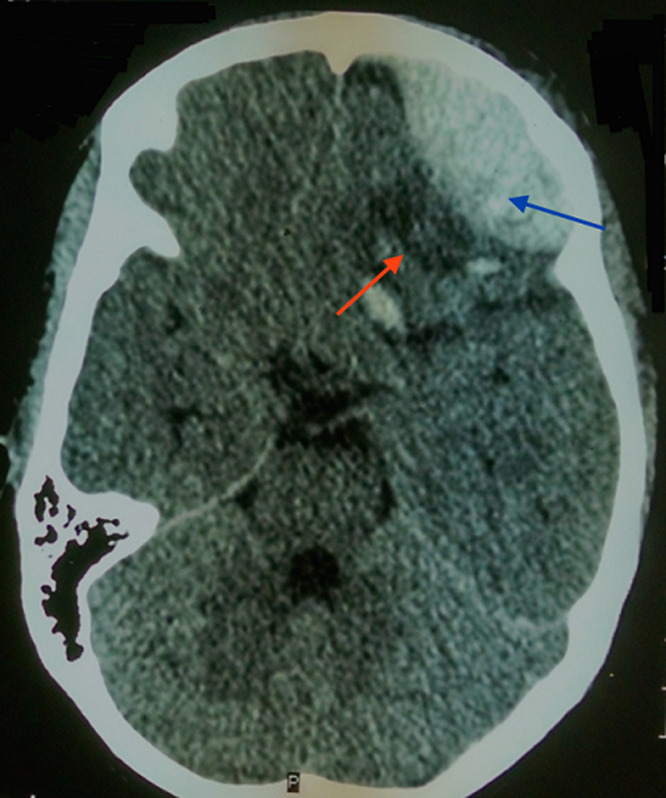
scanner cérébral en coupe axiale montrant l´hématome extradural frontal gauche (flèche bleue), et la contusion sous-jacente (flèche orange)

**Figure 2:**
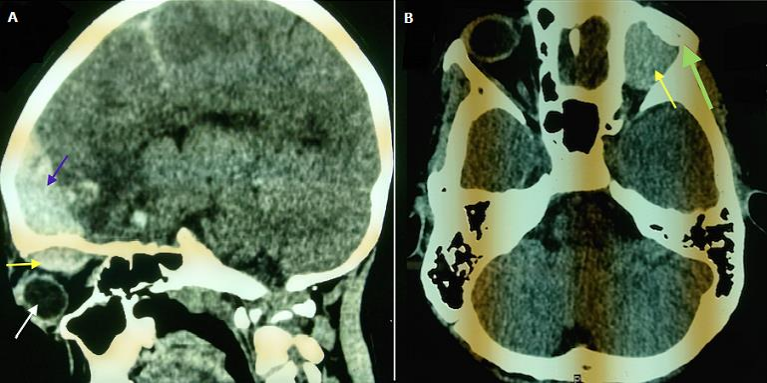
scanner cérébral: A) coupe sagittale: hématome extradural frontal (flèche violette), hématome sous périosté de l´orbite (flèche jaune), globe oculaire (flèche blanche); B) coupe axiale passant par l´orbite: hématome sous périosté (flèche jaune), fracture du rebord orbitaire (flèche verte)

**Sur le plan radiologique:** un scanner cérébral était réalisé en urgence montrant un hématome extradural frontal gauche avec un foyer de contusion oedémato-hémorragique sous-jacent (Figure 1); il s´y associait un hématome sous-périosté de l´orbite gauche refoulant le globe oculaire en bas et en avant ainsi qu´une fracture du rebord orbitaire ipsilatéral (Figure 2). On ne visualisait en revanche aucune fracture du toit de l´orbite. Le bilan biologique ne montrait aucune anomalie.

**Intervention thérapeutique**: une intervention chirurgicale était réalisée en urgence. Une craniotomie frontale gauche nous avait permis l´évacuation de l´hématome extradural en partie liquéfié; aucune fracture du toit de l´orbite n´était observée. La poursuite du décollement de la galea jusqu´au rebord orbitaire nous avait permis de confirmer la fracture de sa partie médio-latérale avec un fragment osseux mobile. Cette orbitotomie fracturaire nous avait facilité l´accès à l´espace orbitaire, puis a évacué l´hématome sous périosté sous forme de sang liquéfié noirâtre mêlé à des caillots. La cavité orbitaire était rincée abondamment et le fragment osseux du rebord orbitaire remis à sa place. Un drain était laissé en sous galéal pendant 48 heures.

**Suivi et résultats**: en post-opératoire immédiat on constatait une régression franche de l´exophtalmie. A J2 post-opératoire l´examen ophtalmologique notait une acuité visuelle 4/10 à gauche et 10/10 à droite. Le fond d´œil était normal. A sa sortie, à J5 post-opératoire, on notait une persistance de l´oedème palpébral supérieur, une régression nette de l´exophtalmie avec une motricité oculaire partielle retrouvée et une disparition de l´inflammation conjonctivale.

**Perspectives du patient**: le patient et son père avaient montré leur reconnaissance pour cette prise en charge qui a permis rapidement une amélioration du patient avec une récupération de la vision et avaient espéré aussi une régression complète des autres signes dans les jours à venir.

**Consentement éclairé du patient**: le consentement éclairé du père et du patient a été obtenu pour une utilisation à but scientifique des données du patient.

## Discussion

L´hématome extradural (HED) intracrânien qui représente environ 0,2 à 6% des complications des traumatismes cranio-encéphaliques est rare dans sa localisation frontale et sous frontale. Les symptômes neurologiques, dans la majorité des cas, sont peu fréquents, ce qui peut faire retarder le diagnostic [1-3]. Au niveau de l´orbite, les hématomes orbitaires sont rares. Ils sont classés selon leur localisation sous-périostée ou intraorbitaire (rétrobulbaire). Ils sont une cause importante d´exophtalmie avec baisse de l´acuité visuelle, pouvant mettre en jeu le pronostic visuel. L´hématome sous-périosté (HSP), le plus souvent post-traumatique, survient essentiellement chez le jeune garçon avec une moyenne d´âge de 17,3 ans [4]. L´association post-traumatique d´un HED frontal et d´un HSP de l´orbite est exceptionnelle, et fréquemment, c´est le scanner cérébral réalisé devant une exophtalmie post-traumatique qui la révèle. A notre connaissance une quinzaine de cas a été rapportée dans la littérature [1-10].

L´hématome sous-périosté de l´orbite post-traumatique se manifeste dans la phase aiguë par une exophtalmie non axiale, non pulsatile, souvent immédiate, avec diplopie. Parfois l´exophtalmie peut apparaitre plusieurs jours après le traumatisme pouvant alors faire retarder le diagnostic. Il s´y associe un déplacement du globe oculaire vers le bas, une ophtalmoplégie, un chemosis, une ecchymose palpébrale, et parfois des dermabrasions cutanées frontales ou un hématome sous-galéal. La présence d´une hémorragie sous-conjonctivale doit faire penser à un hématome rétrobulbaire associé. L´évolution en cas de retard de prise en charge se fait vers la chronicité de l´hématome avec survenue de modifications parfois irréversibles. La baisse de l´acuité visuelle et la cécité en constituent la complication redoutable [1,3,7]. Dans notre cas, le patient présentait tous les signes cliniques évocateurs de l´HSP de l´orbite. Par contre en dehors de la désorientation temporo-spatiale aucun signe n´évoquait une atteinte cérébrale. Les causes de l´HSP de l´orbite sont classées en 4 catégories: traumatique, congestif, systémique et spontané (ou idiopathique). L´HSP post-traumatique est secondaire à un traumatisme direct sur l´orbite ou sur les régions adjacentes. L´hématome sous-périosté congestif survient en général au cours d´un effort responsable d´une congestion veineuse: musculation, plongée sous-marine. Parmi les causes systémiques on peut citer les complications lors des procédures ophtalmologiques ou neurochirurgicales, les troubles de la crase sanguine (coagulopathies, leucémies, anémie falciforme, β-thalassémie), les sinusites chroniques. Quant à l´HSP spontané sans aucun facteur prédisposant il est exceptionnel [1].

On décrit 2 mécanismes dans la survenue de l´HSP post-traumatique qui est le plus fréquent. D´une part, c´est une fracture du toit de l´orbite qui entraine une déchirure des vaisseaux sous-périostés ou une extension d´un HED le plus souvent sous-frontal. Et d´autre part, un hématome sous-galéal dans la région périorbitaire, qui élargit l´espace et s´étend dans l´orbite [1]. Dans notre observation, nous n´avons pas retrouvé de fracture du toit de l´orbite, mais une fracture du rebord orbitaire détachant un fragment orbitaire. Le mécanisme serait à notre avis un saignement par fracture du rebord orbitaire et/ou une extension d´un hématome sous-galéal par élargissement de l´espace sous-galéal au niveau de la fracture. Le diagnostic différentiel se pose devant toute exophtalmie post-traumatique. Il doit alors faire discuter la fistule carotido-caverneuse, la thrombose post-traumatique du sinus caverneux, le corps étranger intra-orbitaire, la compression oculaire par une fracture ou par une fistule artério-veineuse oculaire. Le scanner cranio-cérébral réalisé en première intention en urgence permet le diagnostic positif en montrant l´hématome sous-périosté, l´hématome extradural généralement ipsilatéral et une fracture éventuelle du toit de l´orbite [7,6].

Les modalités du traitement sont controversées. Dans notre cas et dans la plupart des cas similaires, l´intervention chirurgicale par une craniotomie frontale est le Gold standard qui permet l´évacuation de l´HED et une décompression efficace du contenu orbitaire avec recouvrement rapide de la fonction visuelle [1-3,7,10]. Certains auteurs proposent un traitement minimal invasif par aspiration à l´aiguille, notamment en cas d´HSP isolé et/ou associé à un petit HED en phase chronique [8]. Mais cette méthode peut présenter un risque de resaignement ou d´insuffisance d´évacuation de caillots sanguins [1]. Une petite incision sourcilière peut faciliter l´accès à l´espace sous-périosté et constitue une bonne alternative surtout dans la phase chronique [4,9]. Un traitement conservateur peut se concevoir par surveillance seule ou associée aux topiques du maléate de Timolol ou à l´Acétazolamide oral ou en IV; il s´agit des cas d´HSP de petit volume ou d´exophtalmie non progressive sans troubles visuels ni signes d´hypertension intraoculaire. L´essentiel, quelque soit le moyen utilisé, la prise en charge doit être adaptée pour éviter l´évolution vers des séquelles graves: atrophie du nerf optique, strabisme secondaire, lésions cornéennes, exophtalmie persistante par calcification de l´hématome [1].

## Conclusion

L´association d´un hématome extradural frontal et d´un hématome sous-périosté de l´orbite est exceptionnelle. Elle doit être évoquée devant toute exophtalmie post-traumatique. La prise en charge rapide diagnostique et thérapeutique permet d´éviter la survenue de complications.
